# Polymer-Silica Hybrid On-Chip Amplifier with Vertical Pumping Method

**DOI:** 10.1038/s41598-018-31943-z

**Published:** 2018-09-12

**Authors:** Yue Cao, Baizhu Lin, Yue Sun, Yunji Yi, Yijun Liu, Jie Zheng, Fei Wang, Daming Zhang

**Affiliations:** State Key Laboratory of Integrated Optoelectronics, College of Electronic Science & Engineering, Jilin University, Changchun, 130012 P. R. China

## Abstract

This article demonstrates a multilayer polymer-silica hybrid on-chip amplifier combining mode division multiplexing method. The multilayer amplifier consists of a pumping silica waveguide and an amplifying polymer waveguide. The pumping waveguide possesses the stability and the high damage threshold. The amplifying waveguide takes the advantages of the high compatibility and the high doping rate. The vertical pump of mode division multiplexing method can introduce the pumping light into the amplifying waveguide at any desired position of the chip. By the isolation method between signal and pumping light, the pumping light can be coupled into the amplifying waveguide, while the signal light cannot be coupled into the pumping waveguide. The parameters of doping rates, waveguide lengths, overlap factors, coupling parameters are calculated to optimize the gain characteristics of the amplifier. The amplifier with three position-optimized pumping light was designed achieving a maximum gain of 33.89 dB/cm with a waveguide length of 6 cm, a signal power of 0.1 mW and a pumping power of 300 mW. This polymer-silica hybrid amplifier is promising for the on-chip loss compensation of the 3D photonic integrated circuits and all optical transistors.

## Introduction

In modern telecommunication systems, signal light is used to transport information in the transverse directions between different users. The signal light possesses losses such as absorption losses, scattering losses and coupling losses during transportation in the optical devices. Waveguide amplifiers provide a feasible solution to the loss problem in telecommunication systems^1–4^. Waveguide amplifiers include inorganic amplifier and organic amplifier. Inorganic amplifiers consist of the material of LiNbO_3_, Si nanoclusters, rare-earth doping Al_2_O_3_, and rare-earth doped glass exhibiting the gain of 1–7 dB and have stable performance^5–7^. Polymer optical amplifiers doped with high rare-earth ion realise a low-cost device and gives rise to the gain characteristics. And different doping methods and ion-doping rates will affect the amplifying gain performance^8,9^. The gain of polymer amplifier was mainly between 2–15.1 dB^10,11^.

However, the discrete waveguide amplifiers are not suitable for the ultra-long device (such as delay line chips) and highly integrated chips (such as three dimensional photonic integrated circuit (3D PIC) and optics electronic integrated circuit (OEIC))^12–15^. It is because that gain performances of the discrete waveguide amplifiers are restricted by the pumping method. The pumping light of optical waveguide amplifiers is usually coupled at the input facet of signal waveguide. The excess pumping light is absorbed by the active element at the beginning of the waveguide, resulting in an exponential attenuation in pumping power in the signal waveguide. In order to maintain sufficient pumping power over the entire length of the signal waveguide, many different pumping methods and multilayer waveguide structures have been proposed. The pumping light can be gradually introduced in amplifying waveguide and effectively increase the transmission distance of pumping light (such as the planar dual-waveguide coupling, hybrid single layer amplifier and multi-architectural amplifier ect). A two-dimension distributed coupling method of the Nd^3+^-doped polymer waveguide has been used to solve the end face pumping problem^16^. However, for the two-dimension structure, the material of signal waveguide was the same as the pumping waveguide due to the planar fabrication process. What’s more, the transmission isolation between signal light and pumping waveguide can not be realized if signal light wavelength is closed to pumping wavelength. In 2010, a hybrid waveguide comprising optical glass and active Erbium (Er^3+^)-doped phosphate glass has been introduced and achieved over 3 dB/cm gain^17^. And in 2015, a multilayer hybrid phosphate planar waveguide co-doped with Er^3+^–Yb^3+^ ions optimized gain up to 4 dB/cm^18^. These structures are appropriate to the glass amplifier, because the structure of two cohesive waveguide cores can be easily fabricated by the ion-exchange technology. However, for the polymer and silica waveguides with step index distribution, the core cohesive needs additional complex fabrication process. In addition, the two waveguide with cohesive structures have the problem of pumping light leakage from amplifying waveguide to pumping waveguide. Another pumping light coupling method was grating assist coupling. Waveguide structures with grating can obtain high coupling efficiency in integrated optical circuit^19–21^. However, the grating couplers in amplifying field are limited because of its complex experimental process and high cost.

Recently, the optical coupling of two waveguides with a spacing between the centers of the waveguide can be achieved by phase matching of the modes (the effective refractive index equal) of the two parallel waveguides in the mode division multiplexing area^22^. In this study, the phase mismatch between the base mode of signal light and the pumping light, preventing the signal from leaking into the pump waveguide, could realize the isolation of signal light and pumping waveguide. The vertical pumping of mode division multiplexing method can introduce the pumping light into the amplifying waveguide at any desired position of the chip solving the limitation of the end face pumping method. This vertical pumping method is suitable for the silica and polymer waveguide system. We designed a dual-mode hybrid Er^3+^-Yb^3+^ codoped waveguide amplifier composed of pumping silica waveguide and amplifying polymer waveguide in vertival direction in order to facilitate the application of the amplifier to 3D hybrid integrated circuit in multicomponent material system. Firstly, the hybrid structure takes advantage of the high compatibility and high doping rate of the polymer and the stability and high damage threshold of the silica. Secondly, this hybrid integration is especially suitable for the low temperature stability gain material (such as perovskite and quantum dots) owing to the low temperature fabrication process of polymer waveguide such as photobleaching, UV imprinting and 3D printing. Thirdly, polymer-silica hybrid integration possesses low cost and low power consumption during the thermal tuning.

## Design and Simulation

### Structure and design

**Figure 1:**
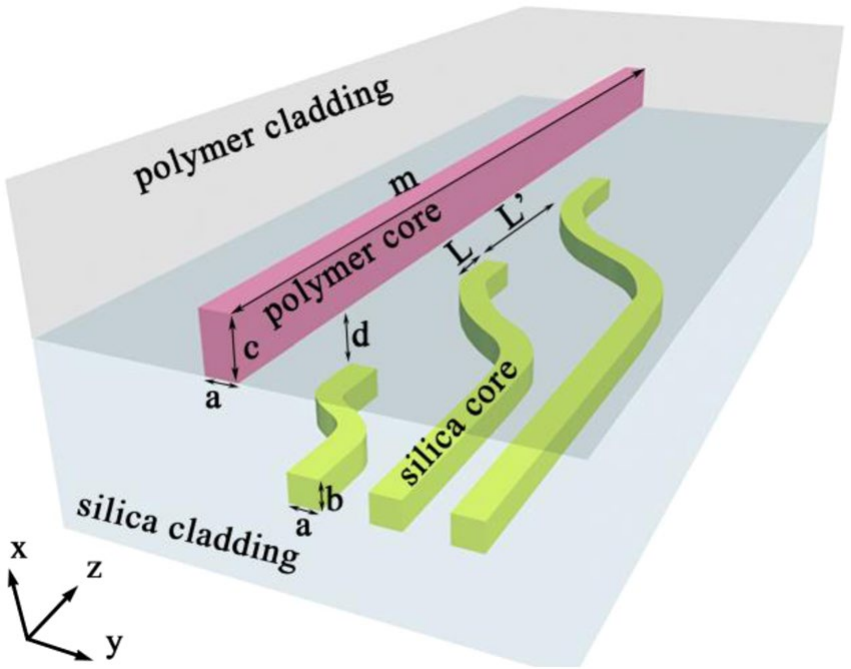
three dimensional schematic of polymer–silica hybrid on-chip amplifier (d = coupling gap; L = coupling length; L’ = pumping light introduction position; m = the length of polymer waveguide).

The hybrid amplifier consists of a pumping silica waveguide layer and amplifying polymer waveguide layer. The schematic of the polymer-silica hybrid optical waveguide amplifier is shown in Fig. 1. The pumping layer waveguide is a single-mode silica waveguide (with the waveguide dimension of a μm × b μm) at 980 nm and 1550 nm wavelengths. Silica material possesses a good tolerance to the high pumping power. The amplifying waveguide consists of a dual-mode polymer waveguide (with the waveguide dimension of a μm × c μm) for the double mode waveguide of 980 nm and a single mode waveguide at 1550 nm. The pumping layer waveguide is designed as a curved structure for the segmenting coupling with the silica amplifying waveguide at the waveguide length of 980 nm. The silica waveguide core is germanium (Ge)-doped silica, and the refractive index is 1.4575 at 980 nm and 1.4515 at 1550 nm^23^. For compatibility with the refractive index of the silica core, the refractive index of the polymer and silica cladding is 1.45 at 980 nm and 1.444 at 1550 nm, respectively (take NOA (Norland Optical Adhesive) and PMMA (Polymethylmethacrylate) for example).

### Signal light and pumping light isolation

We apply signal light and pumping light isolation of the optical amplifier achieving coupling of the 980 nm pumping light (between the polymer waveguide and the silica waveguide) and realizing the independent propagation of 1550 nm (in the polymer waveguide). The effective index of the base mode of the silica waveguide was designed to be identical with the first mode of the polymer waveguide at the wavelength of 980 nm. In addition, the base mode of the polymer waveguide could not couple with the silica waveguide at the waveguide length of 1550 nm because of the mismatch of the effective index.

**Figure 2:**
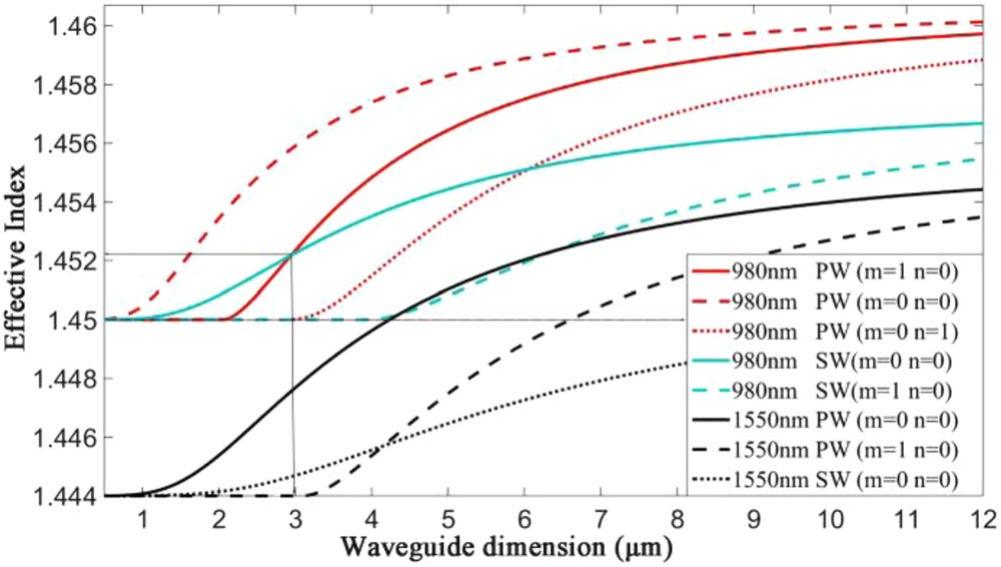
The relationships between the mode index and the waveguide dimensions (PW = polymer waveguide; SW = silica waveguide).

The pumping waveguide is optically isolated from the signal waveguide due to optimization of the length-width ratio (R = c/a), polymer core dimensions and the material refractive index. The relationships between the mode characteristics and the waveguide dimensions are simulated in Fig. 2. Particularly, the length-width ratio of amplifying polymer waveguide is 5.7/3, and a reasonable explanation will be provided later. The intersection of the curves of the base mode of the silica waveguide at 980 nm and the first mode of the polymer waveguide at 980 nm are the optimized effective index value and the waveguide dimensions, respectively.

**Figure 3:**
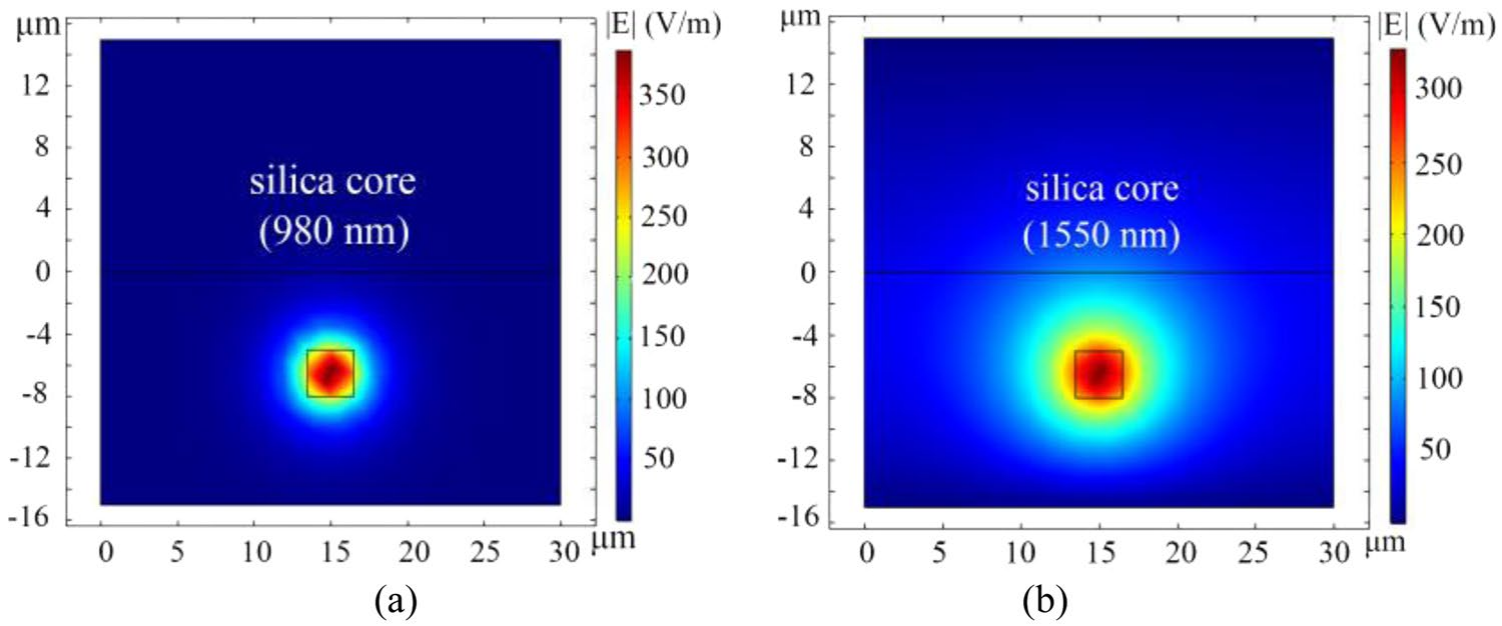
Optical fields distribution of the silica waveguide (y-x plane). (**a**) the base mode optical field of 980 nm in the silica waveguide, (**b**) the base mode optical field of 1550 nm wavelength in the silica waveguide.

**Figure 4:**
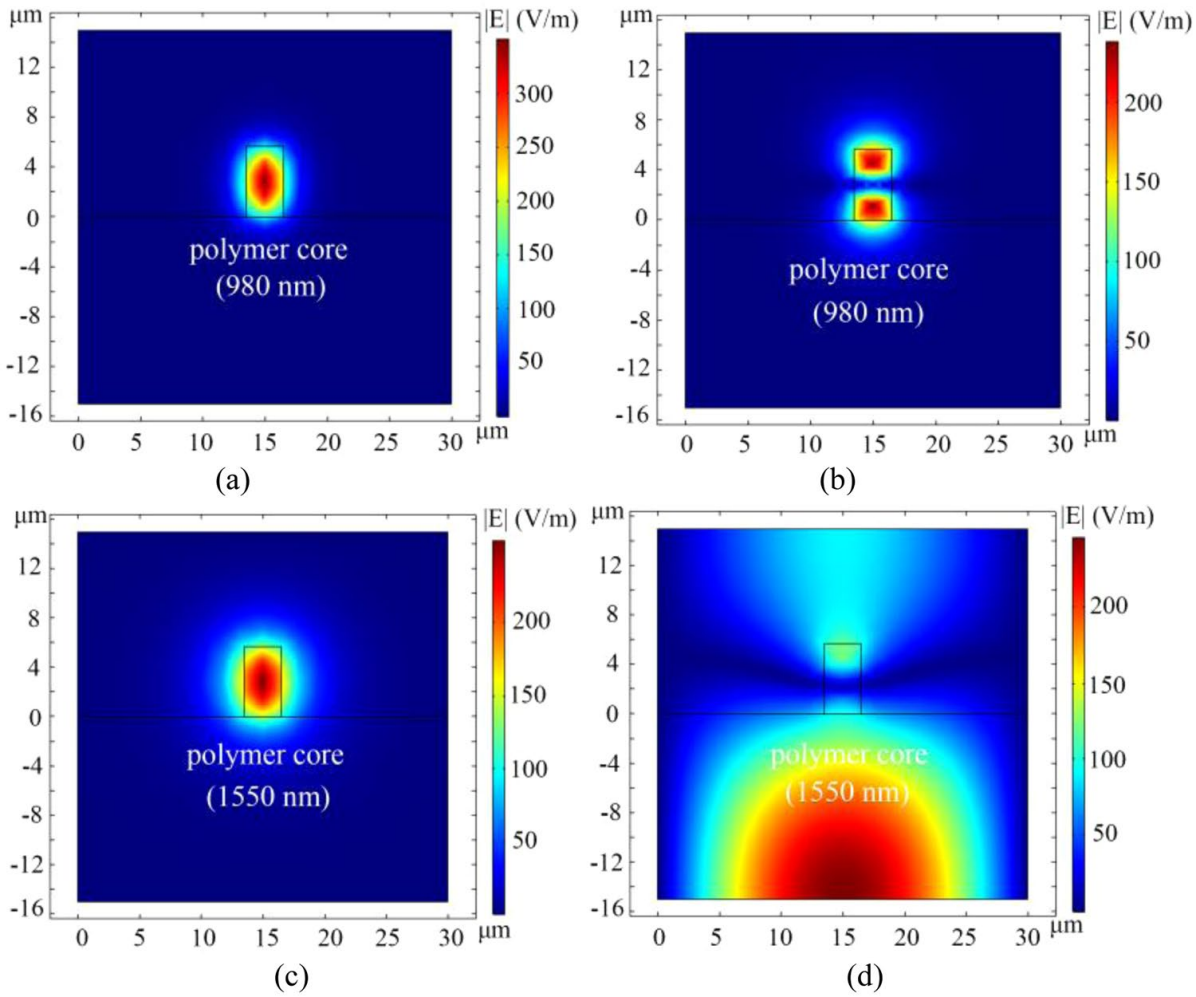
Optical fields distribution of the polymer waveguide (y-x plane). (**a**) the base mode optical field of 980 nm wavelength in the polymer waveguide, (**b**) the first mode optical field of 980 nm wavelength in the polymer waveguide, (**c**) the base mode optical field of 1550 nm wavelength in the polymer waveguide, (**d**) the first mode optical field of 1550 nm wavelength in the polymer waveguide.

Finite-difference method was introduced to simulate the optical fields of the hybrid structure. The base mode optical fields of silica waveguide at 980 nm and 1550 nm are shown in Fig. 3, and the base mode and the first mode of the optical fields of the polymer waveguide at 980 nm and 1550 nm are shown in Fig. 4.

**Figure 5:**
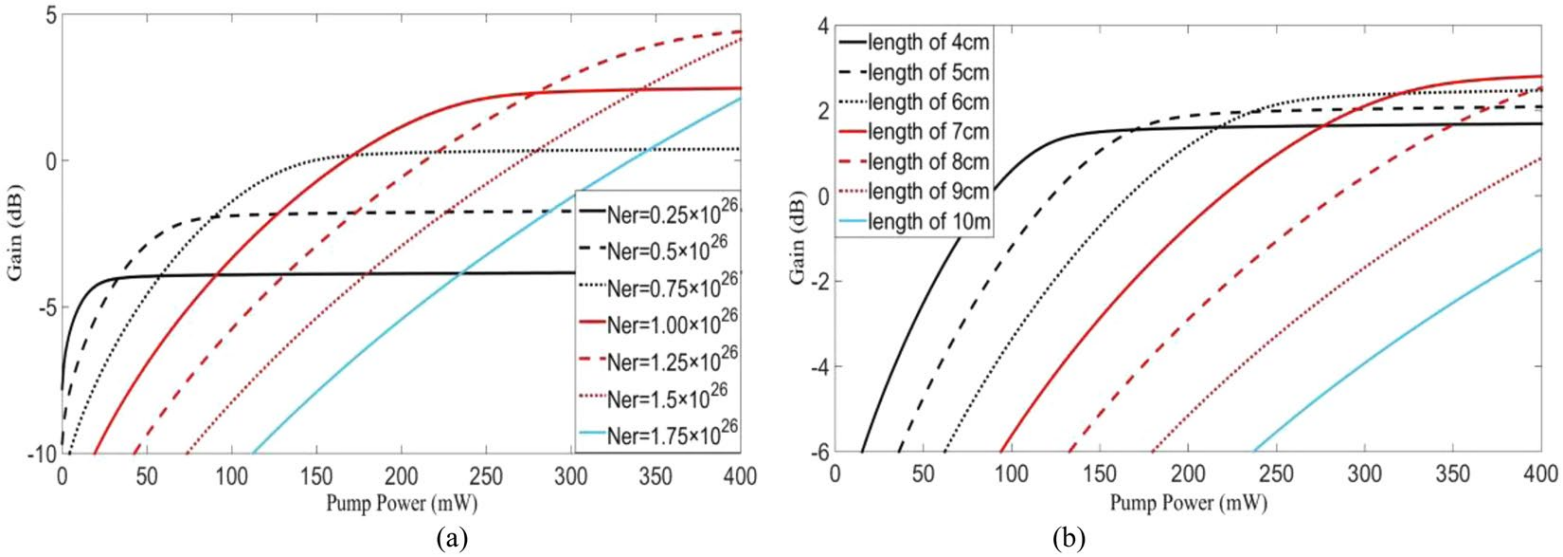
The gain characteristics of the hybrid waveguide. (**a**) the curves of gain and pumping power with different Er^3+^ concentration (the length of the waveguide is 6 cm); (**b**) the curves of gain and pumping power with different waveguides (Er^3+^ concentration is 1.00 × 10^26^).

The gain characteristics of the hybrid waveguide amplifier are simulated by a six-level system model for Er^3+^–Yb^3+^ codoped waveguide amplifier^12^. Gain characteristics with different Er^3+^ concentration of the hybrid waveguide are shown in Fig. 5(a) with the pumping power changing from 100 mW to 300 mW. Thus, the polymer amplifier would have a higher gain by increasing the doping rate.

However, the polymer waveguide amplifier still suffers from the damage threshold of the polymer material. For the end face pumping method, with the fixed propagation loss of 1 dB/cm (it can be optimized for 0.1 dB/cm in experiments) and the fixed ion-doping rate (1.00 × 10^26^), the gain characteristics are related to the pumping power and waveguide length. The gain characteristics are shown in Fig. 5(b). Considering the threshold power of the polymer material, the pumping power should be no more than 300 mW. The maximum device length should be no more than 6 cm. 1.66 dB and 0.46 dB gains can be achieved by a single amplifier with lengths of 4 cm and 8 cm, respectively, when the end face pumping power is 300 mW. However, 3.02 dB gains can be obtained with two amplifiers with only 4 cm length when the end face pumping power is 150 mW. In this respect, the segmenting coupling method would be an effective method to increase the gain characteristics, and the vertical segmenting coupling method on-chip would solve the loss problem of 3D-PIC.

The pumping light was coupled into the signal waveguide in the optimized position on-chip instead of the end face. For the segmenting coupling method, the following discussion mainly emphasizes three factors including the overlap factors, coupling gap, and the pumping light introduction position.

### Overlap factors

The gain characteristics of the mode amplification are mainly determined by the modified overlap factors (Γ′_*s*_) affected by two parameters including the overlap factors between signal light and pumping light (Γ_*sp*_) and the overlap factors of signal light in the entire waveguide (Γ_*s*_). The signal light is transmitted as the base mode at 1550 nm in the polymer waveguide and the pumping light is transmitted as the first mode at 980 nm in the polymer waveguide. The influence of overlap factors of pumping light (Γ_*p*_) on gain characteristics can be neglected. The overlap factors are calculated by the formulas below.

1 $${\Gamma }_{sp}=\frac{{\iint }_{A}{\phi }_{p}(x,y){\phi }_{s}(x,y)dxdy}{\sqrt{{\iint }_{A}{\phi }_{p}^{2}(x,y)dxdy{\iint }_{A}{\phi }_{s}^{2}(x,y)dxdy}}.$$

2 $${\Gamma ^{\prime} }_{s}={\Gamma }_{s}\times {\Gamma }_{sp}.$$

3 $${{\rm{\Gamma }}}_{{\rm{s}}}=\frac{{\iint }_{{\rm{A}}}{{\rm{\phi }}}_{{\rm{s}}}({\rm{x}},{\rm{y}})\mathrm{dxdy}}{{\iint }_{{\rm{\Omega }}}{{\rm{\phi }}}_{{\rm{s}}}({\rm{x}},{\rm{y}})\mathrm{dxdy}}$$

Where “A” is the area of the signal polymer core region. “ Ω” presents the entire amplifying optical waveguide region.

**Figure 6:**
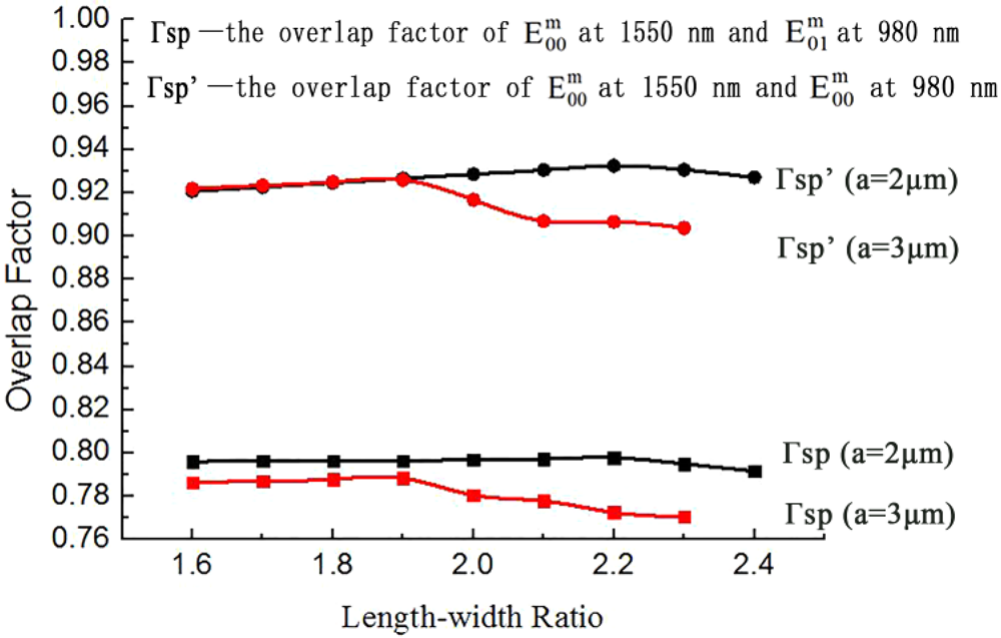
The overlap factors of the hybrid waveguide with different length-width ratio and waveguide dimension (The black line represents the overlap factors with a = 2 μm, the red line represents the overlap factors with a = 3 μm).

Firstly, the overlap factor of the signal light and the pumping light (Γ_*sp*_) is affected by the length-width ratio (R), core optical waveguide dimension, and the refractive index of material. The calculated values of Γ_*sp*_ and (the overlap factors of the base mode at 1550 nm and the base mode at 980 nm wavelength in polymer core layer with the width of a_1_ = 2 μm and a_2_ = 3 μm, respectively) are shown in Fig. 6. For the overlap factors of the base mode at 1550 nm and the base mode at 980 nm wavelengths, optical field center of 1550 nm and 980 nm is almost overlapped for the length-width ratio between 1.6 and 1.9. The effect of waveguide core width on overlap factors can be neglected (Γ′_*sp* a = 2 _≈ Γ′_*sp* a = 3_). With the length-width ratio between 1.9 and 2.3, The increase of the waveguide core width reduces the overlapping area of the light field center of 1550 nm and 980 nm. Thus, the overlap factors will decrease with the increase of waveguide core width at the same overlap factors of 1550 nm and 980 nm (Γ′_*sp* a = 2_ > Γ′_*sp* a = 3_). For the overlap factors of the base mode at 1550 nm and the first mode at 980 nm wavelengths, the light field centers are not overlapped. The effect of the length-width ratio on the overlap factor is the same with the base mode pumping method at 980 nm. Meanwhile, the large waveguide core width will reduce the overlapping area (Γ_*sp* a = 2_ > Γ_*sp* a = 3_). Because the overlap factors will decrease with the increase of the length-width ratio and waveguide core width. With a polymer waveguide width of 2 μm and a length-width ratio of 2.2, a refractive index of polymer core of 1.4635 at 980 nm and 1.4585 at 1550 nm, the optimized values Γsp and Γsp’ are 0.7978 and 0.9324, respectively. Similarly, With a polymer waveguide width of 3 μm and a length-width ratio of 1.9, a refractive index of polymer core of 1.4607 at 980 nm and 1.4557 at 1550 nm, the optimized values Γsp and Γsp’ are 0.7883 and 0.9259 respectively.

The overlap factor of the signal light Γ_*s*_ can also influence the gain characteristics of the hybrid waveguide when its dimension and refractive index are changed. Thus, we need to shed light on the modified overlap factors Γ′_*s*_( = Γ_*s*_ × Γ_*sp*_) to obtain a maximum gain. The calculated parameters of Γ_*s*_, Γ_*p*_(The overlap factor of the pumping light), and the modified overlap factors Γ′_*s*_ with different widths of silica core are shown in Tables 1 and 2, respectively. It is demonstrated that a larger waveguide dimension (a = b = 3 μm) can achieve higher modified overlap factors (Γ′_*s*_ = 0.4736). the refractive index of silica core n_1_ is1.4575 at 980 nm.

**Table 1:** The Calculated Overlap Factors of The Hybrid Waveguide (a1 = b1 = 2 μm).

a_1_ = b_1_	c	R	n_2_ (980 nm)	Γ_*s*_	Γ_*p*_	Γ′_*s*_
2 μm	3.2 μm	1.6	1.4705	0.4338	0.3028	0.3453
2 μm	3.4 μm	1.7	1.4688	0.4425	0.3481	0.3523
2 μm	3.6 μm	1.8	1.4675	**0.4606**	0.3979	**0.3668**
2 μm	3.8 μm	1.9	1.4663	0.3813	0.2988	0.3037
2 μm	4.0 μm	2.0	1.4652	0.3950	0.3445	0.3148
2 μm	4.2 μm	2.1	1.4643	0.4103	0.3860	0.3272
2 μm	4.4 μm	2.2	1.4635	0.4249	0.3860	0.3390
2 μm	4.6 μm	2.3	1.4629	0.3227	0.3007	0.2565
2 μm	4.8 μm	2.4	1.4621	0.3313	0.3190	0.2622

**Table 2:** The Calculated Overlap Factors of The Hybrid Waveguide (a2 = b2 = 3 μm).

a_2_ = b_2_	c	R	n_2_ (980 nm)	Γ_*s*_	Γ_*p*_	Γ′_*s*_
3 μm	4.8 μm	1.6	1.4626	0.5590	0.5842	0.4396
3 μm	5.1 μm	1.7	1.4619	0.5823	0.6182	0.4583
3 μm	5.4 μm	1.8	1.4612	**0.6012**	0.6439	**0.4736**
3 μm	5.7 μm	1.9	1.4607	0.5380	0.6079	0.4241
3 μm	6.0 μm	2.0	1.4601	0.5542	0.6289	0.4326
3 μm	6.3 μm	2.1	1.4598	0.5778	0.6578	0.4494
3 μm	6.6 μm	2.2	1.4593	0.5916	0.6738	0.4570
3 μm	6.9 μm	2.3	1.4590	0.5011	0.6120	0.3861

a = the width of core; b = the height of silica core; c = the height of polymer core; n_2_ = the refractive index of polymer core; R = length-width ratio (The refractive index of n_2_ is reduced by approximately 0.006 at 1550 nm).

**Figure 7:**
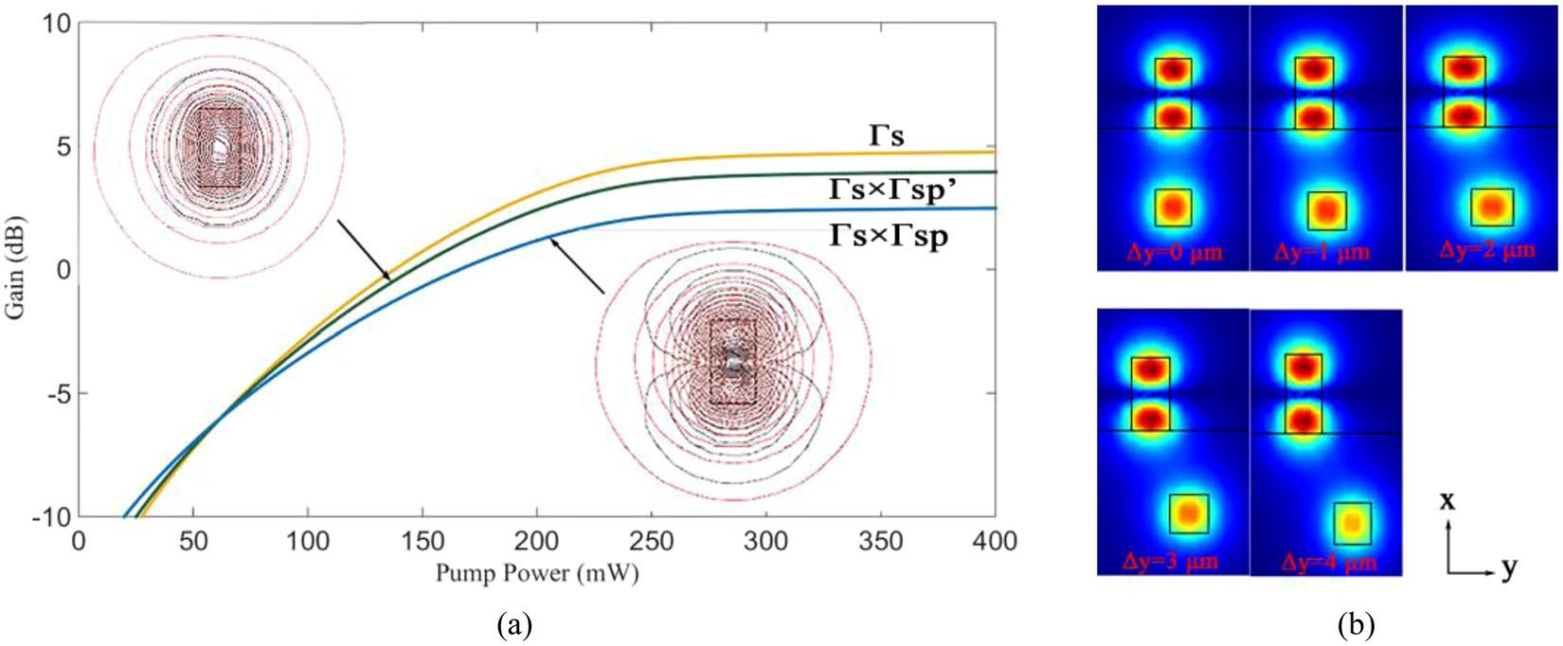
(**a**) The relationships between the pumping power and the gain (The red lines represent the equivalent line of 1550 nm light field in polymer waveguide; The black lines represent the equivalent line of 980 nm light field in polymer waveguide). (**b**) Optical fields distribution of the polymer waveguide of the first mode with changing of the horizontal offset between the waveguides (Δy) (d = 5 μm).

The relationships between the pumping power and the gain are shown in Fig. 7(a). The base mode pumping method and the first mode pumping are comparable. Both of them are feasible solution to the loss problem. The green line and blue line represent the gain characteristics of considering the influence of the overlap factor of Γ_*sp*_ and Γ′_*sp*_ on the overlap factor of signal light Γ_*s*_ respectively. Figure 7(b) Shows the optical fields distribution of the first mode in polymer waveguide with changing of the horizontal offset between polymer waveguides and silica waveguide (Δy). The effective refractive index of the first mode in polymer waveguide is equal to the base mode in silica waveguide. We observed that the influence of the Δy on optical fields distribution of the first mode and overlap factors can be neglected.

### Coupling gap and coupling length

**Figure 8:**
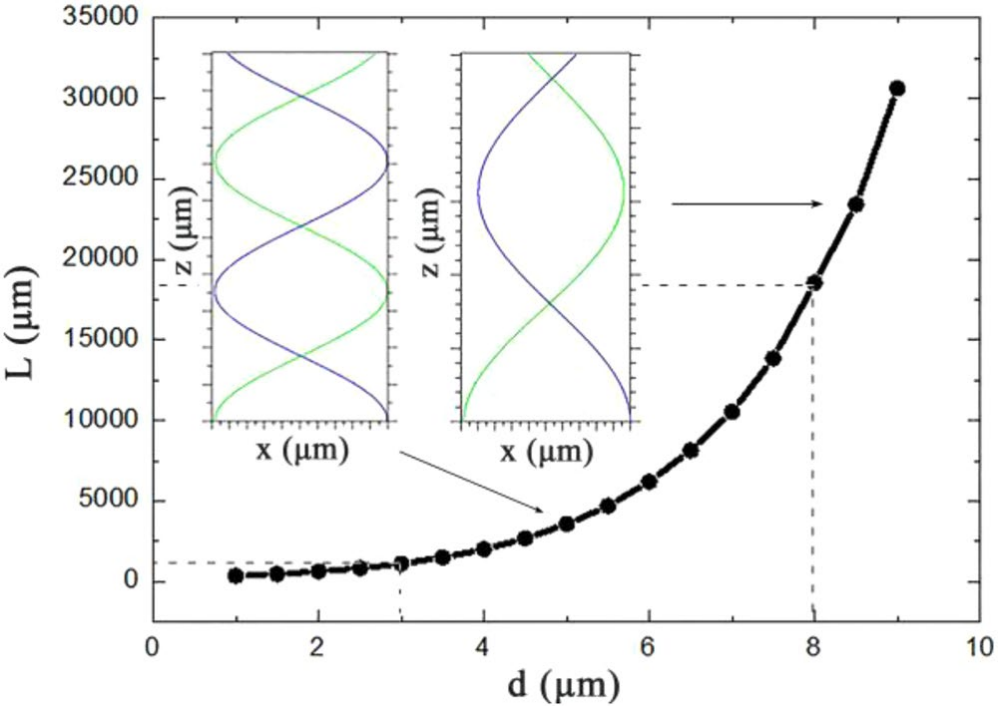
The coupling length with different coupling gap.

**Figure 9:**
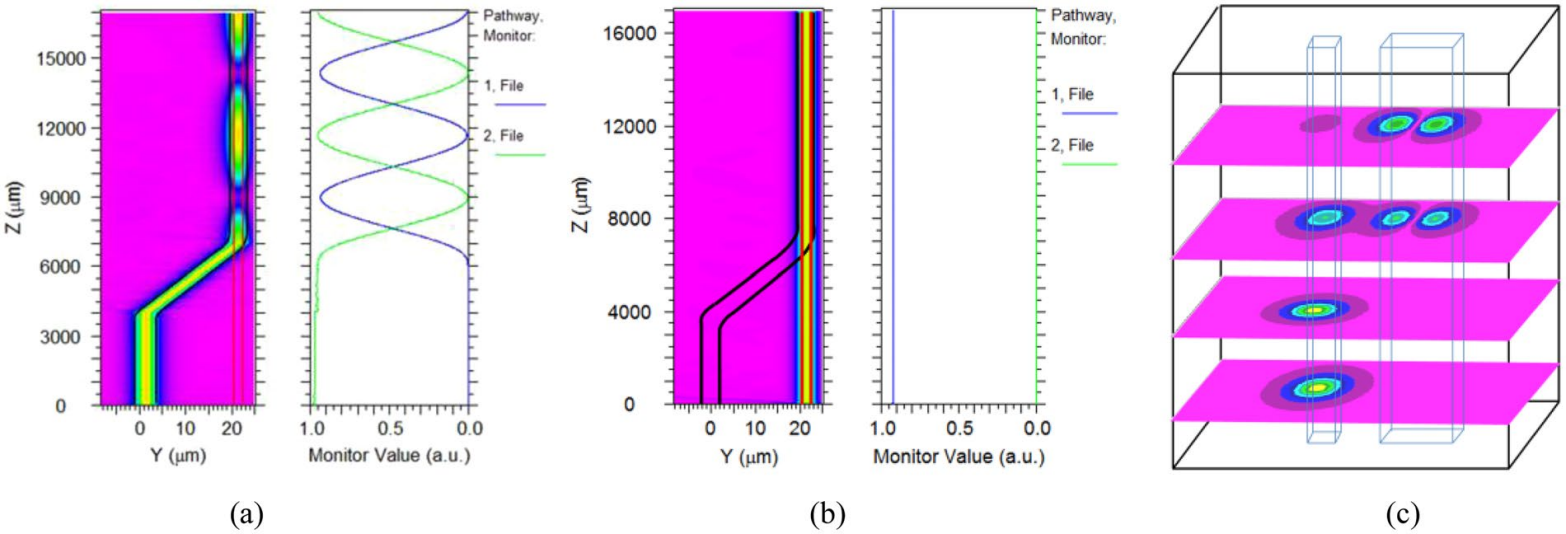
The simulation of optical fields of the two waveguides. (**a**) The propagation and coupling curves of the two waveguides in y-z plane (d = 5 um) when pumping light is introduced from silica waveguide; (**b**) The propagation and coupling curves of the two waveguides in y-z plane (d = 5 um) when signal light is introduced from polymer waveguide; (**c**) the propagation of the base mode and the first mode along the hybrid waveguide in x-y plane when pumping light is introduced from silica waveguide.

It is necessary to optimize the coupling gap to realize the complete coupling of the first mode at 980 nm. Fig. 8 shows that the relationships between the coupling length and the coupling gap. The coupling curves on the left (d = 5 μm) and the right (d = 9 μm) in the coupling region of optical waveguide are shown in Fig. 8 respectively (neglecting the bending loss). The pumping waveguide length (L) should be precisely controlled preventing the back coupling of pumping light. The optical field coupling of the two waveguides is shown in Fig. 9. When the pumping light was introduced from the silica waveguide, as shown in Fig. 9, the base mode of the silica waveguide at 980 nm could be coupled (coupling ratio >98%, 3 um <d <8 um) into the first mode of polymer waveguide. However, when the signal light was introduced from the polymer waveguide, the base mode of polymer waveguide at 1550 nm could not be coupled into the silica waveguide.

### Pumping light position

Finally, the effect of the pumping light position (L′) has been analyzed. The gain characteristics are affected by pumping power, signal waveguide loss and pumping waveguide loss.

**Figure 10:**
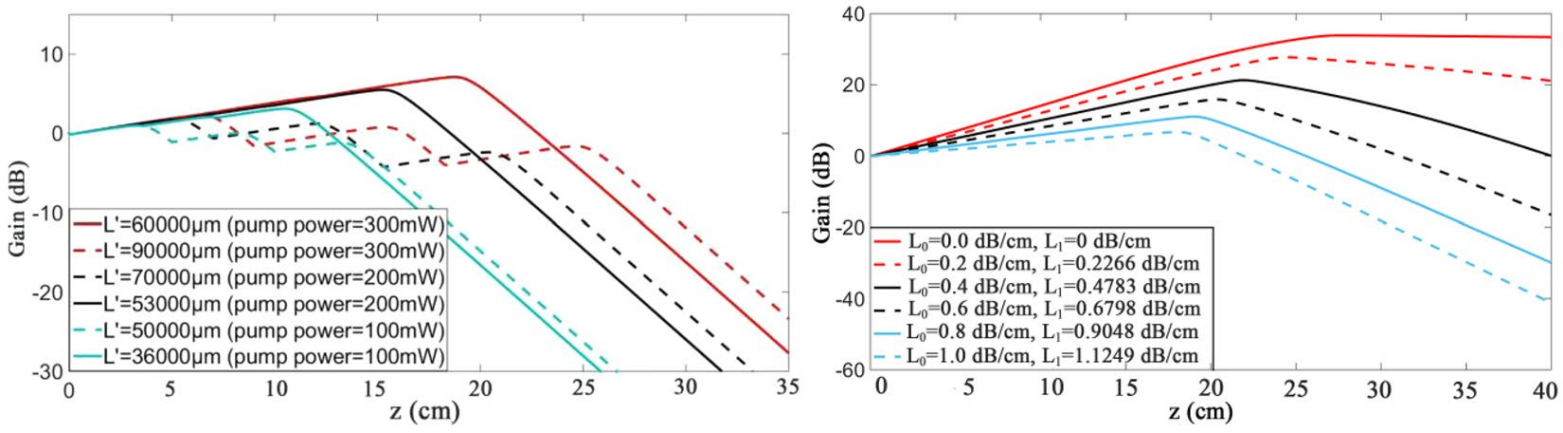
The relationship between gain and pumping light introduction position. (**a**) with different pumping power (L_0_ = 1 dB/cm); (**b**) with different mode losses in waveguide.

The characteristics of the gain of the segmenting coupling waveguide are shown in Fig. 10(a). Because of the dual vertical coupling method, high pumping efficiency and any desired pumping position can be realized. The pumping light is gradually introduced to the signal waveguide in the position where the gain began to drop. The gain peak and pumping light position (L′) is in proportion to the pumping power. The optimized gain characteristics of the hybrid on-chip amplifier could be up to 7.103 dB with a length of 6 cm, and a pumping power of 300 mW.

The gain characteristics of the hybrid waveguide with different base mode loss (L_0_) in polymer waveguide at 1550 nm and the first mode loss (L_1_) in polymer waveguide at 980 nm are shown in Fig. 10(b). The results confirm that a lower transmission mode losses enhance the effect on the peak and growth of gain in the amplifier. The maixmum gain characteristics of the 3D hybrid on-chip amplifier is 33.89 dB/cm with a waveguide length of 27.68 cm, a pumping spacing of 6 cm, the three pumping light introduced position of 0.01 cm, 6.01 cm, and 12.01 cm respectively, a signal power of 0.1 mW, and a pumping power of 300 mW.

## Conclusion and Discussion

In this article, we designed a polymer–silica hybrid on-chip amplifier for 3D-PIC with vertical pumping of mode division multiplexing methods. The Er^3+^-doping rate is 1.00 × 10^26^; the waveguide lengths is 6 cm; The modified overlap factors (Γ′_*s*_) is 0.4736; The coupling gap (d) is 5 um. Ultimately, the maixmum gain characteristics of the 3D hybrid on-chip amplifier is 33.89 dB/cm with a pumping light introduced position of 6 cm, a signal power of 0.1 mW, a pumping power of 300 mW. The results indicate that the 3D coupling pumping method not only increases the gain characteristics, but also solves the threshold of the polymer amplifier and the limitation of the end face pumping method. This polymer-silica hybrid amplifier is promising for the on-chip loss compensation of the 3D photonic integrated circuits.
